# Transportation Mode Detection Using an Optimized Long Short-Term Memory Model on Multimodal Sensor Data

**DOI:** 10.3390/e23111457

**Published:** 2021-11-03

**Authors:** Ifigenia Drosouli, Athanasios Voulodimos, Georgios Miaoulis, Paris Mastorocostas, Djamchid Ghazanfarpour

**Affiliations:** 1 Department of Informatics and Computer Engineering, University of West Attica, 12243 Athens, Greece; gmiaoul@uniwa.gr (G.M.); mast@uniwa.gr (P.M.); 2 Department of Informatics, University of Limoges, 87032 Limoges, France; ghazanfarpour@unilim.fr

**Keywords:** transportation mode detection, deep learning, recurrent neural networks, LSTM

## Abstract

The advancement of sensing technologies coupled with the rapid progress in big data analysis has ushered in a new era in intelligent transport and smart city applications. In this context, transportation mode detection (TMD) of mobile users is a field that has gained significant traction in recent years. In this paper, we present a deep learning approach for transportation mode detection using multimodal sensor data elicited from user smartphones. The approach is based on long short-term Memory networks and Bayesian optimization of their parameters. We conducted an extensive experimental evaluation of the proposed approach, which attains very high recognition rates, against a multitude of machine learning approaches, including state-of-the-art methods. We also discuss issues regarding feature correlation and the impact of dimensionality reduction.

## 1. Introduction

As urbanization and populations have increased in recent years, transportation has become a major factor that affects the experience of people living in big cities. Quality of life, urban productivity, energy consumption, congestion, the environment, and personal safety—which are highly connected with citizens’ mobility—are all affected. The knowledge that can be gained from observing human mobility models in different transport environments can make a significant contribution to addressing these issues in the most appropriate manner. Citizens’ transportation involves multiple transportation modes that change over time, depending on users’ needs.

With the recent the growth of the Internet of Things (IoT), several attempts have been made in the development of new methods to observe urban mobility by using APC systems (APCSs). The operation of these systems is based on Wi-Fi access points (APs) [1], infrared sensors [2,3], video image sensors [4,5], etc. With the advent of modern mobile devices such as tablets, smartphones, and smartwatches, new efficient and user-centric ways have been discovered [6] to detect an individual citizen’s movements throughout the city and collect detailed data about their journey map. 

Transportation mode detection (TMD), often regarded as a subfield of the activity recognition field, aims to identify the means of transport used by an individual. TMD is important for many reasons that concern the collection of data for urban transportation planning, sending targeted advertisements to users, developing context-aware applications, urban planning and traffic management, physical and mental health improvement, promotion of soft transportation modes (walking, running, and biking), bicycle usage planning, etc. [7].

With the advancement of sensing technologies, modern mobile devices such as smartphones, smartwatches, tablets, etc., can be involved at many points in the process of transportation mode detection, by collecting and analyzing multimodal sensor data in the field of transportation. Modern mobile devices now have two important characteristics that make them more than just simple devices:

Firstly, the recent huge technological advances and the various sensors they are equipped with allow them to measure motion, orientation, and environmental conditions continuously, with high precision and accuracy, providing massive data that are available to be used immediately. Furthermore, the fact that 80.76% of the world’s population owns a smartphone [8], that the smartphone adoption among adults aged 50 and older has increased from 62% (2017) to 79% (2019) [9], and that the number of smartphones in use is growing at an annual rate of 5.6% [10], shows that modern mobile devices have become a considerable factor in people’s daily lives in the past decade.

This makes it more possible than before to capture information that, until now, was unreachable, such as where the users are, how much time they spend in certain places, what they are interested in, what they like, and even how they feel. Modern mobile devices’ capabilities, combined with the tremendous advances in deep learning, open new possibilities in the transportation community, and especially in smart mobility. 

In this work, we present a deep learning approach for transportation mode detection using multimodal sensor data elicited from user smartphones. The approach is based on long short-term memory networks and Bayesian optimization of their parameters. We conducted an extensive experimental evaluation of the proposed approach with a multitude of machine learning approaches, including ensemble and deep models. The remainder of the paper is structured as follows: Section 2 offers a brief overview of related works, Section 3 presents the Bayesian-optimized LSTM model. In Section 4, we perform an extensive experimental evaluation of the proposed model compared to a variety of other methods. Finally, Section 5 concludes the paper with a summary of our findings.

## 2. Related Work

In recent years, several models have been developed based on sensors embedded in smartphones (such as accelerometers, magnetometers, gyroscopes, atmospheric pressure, GPS, etc.) so as to detect transportation mode using traditional machine learning as well as deep learning techniques. 

To our knowledge, there are four publicly available datasets concerning transportation modes that citizens use in their everyday lives:

- The first is Microsoft GeoLife’s dataset [11], collected in the (Microsoft Research Asia) GeoLife project by 182 users in a period of over three years (from April 2007 to August 2012), but this contains only GPS data;
- The second is the HTC dataset [12], which includes up to 8311 h of collected data, but only from 3 sensors. This dataset could not be found, even though it is supposed to be public for research;
- The third is the US-TMD dataset [13], which contains 31 h of data. It is based on 13 users who collected the data during their daily activities. The dataset contains 5893 samples, includes all sensors available in phones, and distinguishes 5 transportation modes: car, bus, train, still, and walking;
- The fourth dataset, which is the dataset used in this work, is the Sussex-Huawei Locomotion–Transportation (SHL) dataset, and is considered to be one of the biggest datasets in the research community. It includes 2812 h of annotated data collected for 7 months by 3 participants. Each participant carried 4 smartphones at 4 body locations (hand, torso, hip pocket, and backpack), and 15 sensors in the smartphone were used for recognizing 8 modes of transportation (i.e., still, walk, run, bike, car, bus, train, and subway).

Models: Various traditional (“shallow”) machine learning methods on multimodal sensor data have been used in the field of TMD. In [14], the sensor data were divided into frames with a sliding window size of 5.12 s with half overlap, and in each frame computed seven features from the magnitude of the three motion sensors. For the accelerometer, the mean, standard deviation, index of the highest FFT (fast Fourier transform value), and ratio between the first- and second-highest FFT values were computed. For the gyroscope, mean and standard deviation were computed, while for the magnetometer only standard deviation was computed. A decision tree algorithm was employed to train a transportation mode classification model, achieving a 71% F1-score. In the 2020 Research Challenge [15] created by the University of Sussex and Huawei Ltd., among the ML classifiers used, XGBoost achieved the highest F1-score (77.9%), followed by random forest (69.1%) and multilayer perceptron (MLP) (52.8%). 

Different machine learning classifiers (K-nearest neighbor, support vector machines, tree-based methods, etc.) were developed in [16] to identify different transportation modes, including bike, car, walk, run, and bus, with random forest producing the best overall performance of 95.1%. Tree-based ensemble models (random forest, gradient-boosting decision tree, and XGBoost) were used in [17] to classify the different transportation modes using Global Positioning System (GPS) data. The experimental results showed that the XGBoost model produced the best performance, with a classification accuracy of 90.77%. In [13], three datasets were created and used for TMD, each with different types of sensors. For each of these datasets, four classification machine learning algorithms were used: decision trees (DT), random forest (RF), support vector machines (SVM), and neural network (NN). For all datasets, random forest had the highest accuracy (81–93%).

Numerous experiments were carried out in [18] to compare the impact of different feature sets (e.g., time-domain features, frequency-domain features, Hjorth features), as well as the impact of various classification algorithms (e.g., random forest, naive Bayes, decision tree, K-nearest neighbor, support vector machine), on the prediction accuracy. This system achieved an average accuracy of 98.33% in detecting the vehicle modes when using the random forest classifier.

Deep learning techniques, which attract significant interest in the machine learning community, were applied in addition to traditional ML algorithms to the transportation mode recognition task, in order to improve the models’ performance. A unified framework (CL-TRANSMODE) composed of CNN and LSTM was proposed in [19], using the SHL dataset, and managed to outperform DNN, CNN, RNN, LSTM, decision tree, random forest, AdaBoost, and XGBoost for identifying eight transportation modes with a 98.1% accuracy. In [20], a model to detect transportation modes based on a partially observed sequence was presented, and CNN, LSTM, and DNN were used for comparison; the proposed model outperformed them, with an accuracy of 92%. 

In [21], ML and DL techniques were used on the same dataset as in [13]. Random forest had the best performance among all methods (87%), while convolutional neural network (CNN) and long short-term memory (LSTM) had F1-scores of 80% and 76%, respectively. A series of machine learning approaches for real-time transportation mode recognition were presented in [22], built on both statistical feature extraction and raw data, and a comparison was made for these approaches using random forest (RF), support vector machines (SVMs), feed-forward neural networks (FFNNs), multilayer LSTM recurrent neural networks, recurrent neural networks (RNNs), and convolutional neural networks (CNNs). RNNs obtained the best performance (88%) using statistical features, while CNNs obtained 98.6% via the analysis of the seven raw data measures without any pre-processing. In [23], a deep neural network (DNN)-based approach was proposed to efficiently recognize five transportation modes (still, walk, run, bike, and vehicle) from accelerometer, magnetometer, and gyroscope measurements. The DNN achieved approximately 95% classification accuracy, and outperformed four machine learning methods, i.e., AdaBoost, decision trees (DT), K-nearest neighbors (KNN), and SVM. In [24], a novel input set consisting of extracted features, rather than raw data, was fed to an LSTM model, for 10 different transportation modes, achieving 96.82% performance. A CNN model built on the one-dimensional acceleration data was used to determine the transportation mode in [25]. Different architectures and classification methods (naive Bayes, Bayes network, decision tree, K-nearest neighbor, random forest, adaptive boosting, neural network, supporting vector, and LSTM) were tested. The proposed approach achieved an accuracy of 94.48%.

According to the literature above, although machine learning (including deep learning) techniques have been applied in transportation mode detection using smartphone sensor measurements, often successfully, most of them have investigated a limited number of algorithms. In [12,14,16,18,23], only traditional machine learning approaches were exploited, or ensemble methods in [17], or only DL in [24]. Other works have implemented a still limited selection of both conventional ML and DL techniques [15,19,21,22,25], but the performance attained has room for improvement. In this work, a wide variety of algorithms (e.g., traditional ML algorithms, ensemble methods, DL algorithms) was used as a benchmark for comparison of our proposed Bayesian-optimized LSTM model. Some works have tried to use very few sensors so as to save on energy consumption, such as the accelerometer, gyroscope, magnetometer in [14,23], as well as pressure in [19] and rotation vector in [16] in addition to the basic sensors, only GPS in [17], and only an accelerometer in [20]. However, the information acquired from this limited number of sensors, as well as the limitations associated with the use of GPS sensors (signal losses), resulted in a low prediction accuracy. Our model achieved higher prediction accuracy by using a wider range of sensor measurements (accelerometer, gyroscope, magnetometer, pressure sensor, GPS (altitude metrics), and temperature) so as to enable more accurate prediction. Several classifiers are built on statistical features [13,18,24], and others on raw data [15,22], as input variables to the model. In our work, preprocessing our raw data and then applying feature extraction methods to reduce the dimensional space of data resulted in improved performance.

The goal of this paper was to build a robust and optimized TMD system using sequential information from multiple smartphone sensors, with an increased detection accuracy compared to existing methods. The contributions of this work with respect to the state of the art are as follows: (1) the development of a probabilistic Bayesian-optimized LSTM framework, through which the model configuration parameters are optimally tuned, resulting in outperforming other state-of-the-art methods for identifying all eight transportation modes; (2) the scrutinization of the effectiveness of a large number of conventional machine learning and deep learning methods used for transportation mode detection based on multimodal smartphone sensor data; (3) a first attempt to explore techniques for understanding and visualizing feature maps for features’ correlation impact on the model performance; (4) although time measurements may vary depending on the system and model architecture, numerous experiments were carried out to compare the impact of different features and algorithm parameters on process time. 

## 3. Bayesian-Optimized Long Short-Term Recurrent Modelling for Transportation Mode Detection

### 3.1. LSTM for Recognition of Transportation Mode

Recognition of transportation mode is an inherently recurrent problem; hence, the employment of recurrent neural networks appears to be a natural choice. A strong advantage of recurrent neural networks (RNNs) is their ability to use contextual information when mapping between input and output sequences. Unfortunately, one problem is that the sensitivity of a given input on the hidden layer and, therefore, on the network output, decreases over time as it cycles around the network’s recurrent connections and the network “forgets” the first inputs. This effect is often referred to in the literature as the “vanishing gradient problem” [26]. Numerous attempts were made in the early 1990s to deal with the problem of vanishing gradients for RNNs. One such approach is the long short-term memory (LSTM) architecture [27].

LSTM is a type of recurrent neural network (RNN) that allows the network to retain long-term dependencies at a given time from many timesteps before; it consists of a set of recurrently connected subnets, known as memory cells, which allow the network to store and access information over long periods of time. LSTM can combine simple deep neural network architectures with smart mechanisms so as to learn what parts of history to “remember” and what to “forget” over long periods. The ability of LSTM to learn patterns in data over long sequences makes it suitable for time-series forecasting.

The memory cell of an LSTM network contains three different components: (1) the forget gate, (2) the input node and the input gate, and (3) the output gate. Each component applies a nonlinear relation to the inner product between the input vectors and respective weights (estimated through a training process). Some of the components have the sigmoid function, expressed as σ(·), while others the hyperbolic tangent function, tanh(·).

The forget gate F(n) separates the information that should be retained from the unnecessary information, by keeping the latter out of the memory cell [28]. The input node H(n) appropriately activates the respective state (true or false output from the “tanh” activation). The input gate I(n) regulates whether the respective hidden state is “significant enough” for the accurate estimation of the transportation mode. The output gate O(n) regulates whether the response of the current memory cell is “significant enough” to contribute to the next cell. 

One characteristic of the memory cell of the conventional LSTM is that it processes only previous state information and, thus, cannot always successfully model non-causal phenomena. The causality property indicates that the system output (i.e., in our case, the current mode of transportation) depends solely on past and current inputs, and not future ones, which may not always be the case. Bidirectional LSTM can provide a promising alternative in this case, since it processes data in both directions, including a forward and a backward pass [29], thus accounting for potential dependencies from “future” instances. In the experimental evaluation section we will scrutinize the effectiveness of both the plain and bidirectional LSTM for the problem at hand.

### 3.2. Bayesian Optimization

Regarding the selection of model parameters, in this work we employed a probabilistic Bayesian framework, through which the model configuration parameters were optimally tuned. 

Assuming that a certain number of configuration parameters are available, such as the number of memory cells, the learning rates, etc., denoted as $\pi_{i}$, if we construct a set of Q different configurations—i.e., $D_{1:Q}=\left\{\begin{array}{ccc} \pi_{1} & \cdots & \pi_{Q} \end{array}\right\}$—then we can evaluate the error E(p,d,π) that the network gives when (1) it receives as inputs the data *p*, (2) the network output is compared against the desired (target) outputs *d*, and (3) a given π model configuration. In this context, we have omitted index *n*, since we refer to any time instance. Let us denote as $E_{min}$ the minimum across all *Q* configurations. Then, an improvement function is given:

(1) $$I\left(p,d,\pi )\right)=max\left\{0,E_{min}-E\left(p,d,\pi \right)\right\}$$

In a probabilistic framework, we estimate:

(2) $$Expect\left(I\left(p,d,\;\pi \right)\right)=Expect\left(max\left\{0,E_{min}-E\left(p,d,\;\pi \right)\right\}\right)$$

Equation (2) can be solved only by knowing the probability distribution of the error function given a set of configurations, i.e., $P\left(E|D_{1:Q}\right).$ Based on the Bayesian rule, we have:

(3) $$P(E|D_{1:Q})\propto P\left(D_{1:Q}|E\right)P\left(E\right)$$

P(E) generally follows a Gaussian distribution, and $P\left(D_{1:Q}|E\right)$ is then expressed as a Gaussian process of mean μ(π) and standard deviation Σ [30]:

(4) $$\Sigma =\left[\begin{array}{ccc} k\left(\pi_{1},\pi_{1}\right) & \cdots & k\left(\pi_{1},\pi_{Q}\right)\\ \vdots & \ddots & \vdots \\ k\left(\pi_{Q},\pi_{1}\right) & \cdots & k\left(\pi_{Q},\pi_{Q}\right) \end{array}\right]$$

where k(·) is a kernel function. The goal of the optimization is to find a new configuration $\pi^{*}\equiv {\;\pi }_{Q+1}$, which decreases the MSE or equivalently increases the improvement $I\left(p,d,\pi^{*}\right)$. For the augmented set $D_{1:Q+1}$ containing $\pi^{*}\equiv {\;\pi }_{Q+1}$, $P\left(D_{1:Q+1}|E\right)$ will again be a Gaussian process of standard deviation:

(5) $$\left[\begin{array}{cc} \Sigma & b\\ b^{T} & k\left(\pi_{Q+1},\pi_{Q+1})\right) \end{array}\right]$$

where $b=\left[k\left(\pi_{Q+1},\pi_{1}\right)\ldots k\left(\pi_{Q+1},\pi_{Q}\right)\right]$. It can be proven [22] that $P(E_{Q+1}|D_{1:Q},\;\pi_{Q+1})$ is also Gaussian, with a mean value and standard deviation related to previous variables. Equation (2) can be used to compute the new configuration $\pi^{*}$, as the integral of I(·) and $P(E_{Q+1}|D_{1:Q},\;\pi_{Q+1})$, i.e., the probability that I(·) follows. 

## 4. Experimental Evaluation

This section presents the performance analysis of the proposed Bayesian-optimized LSTM-based model. Firstly, a brief description of the datasets is provided, followed by data preparation and the analysis of the experimental results.

### 4.1. Dataset Description

The SHL dataset used in the experiments is a subset of the original Sussex-Huawei Locomotion–Transportation (SHL) dataset, which contains the data recorded from one participant’s phone placed in their front trouser pocket, and includes a period from 1 March 2017 to 5 July 2017. For the analysis, eight main activities are considered: still, walk, run, bike, car, bus, train, and subway. The number of samples per class are shown in Table 1. SHL is a multivariate time-series dataset that contains 23 features representing measurements from 6 smartphone sensors: accelerometer, gyroscope, magnetometer, pressure sensor, GPS (altitude metrics), and temperature. Even though the number of participants was limited, the focus was on the quality of the collected and annotated data, and on collecting real-life data over a long period (2812 h of labeled data and 17,562 km of traveled distance collected over 7 months) [6].

The data were used to frame a forecasting problem where, given the sensor measurements and mode of transport used previously, the mode of transport at the next time could be predicted.

### 4.2. Preliminary Data Analysis

**Figure 1:**
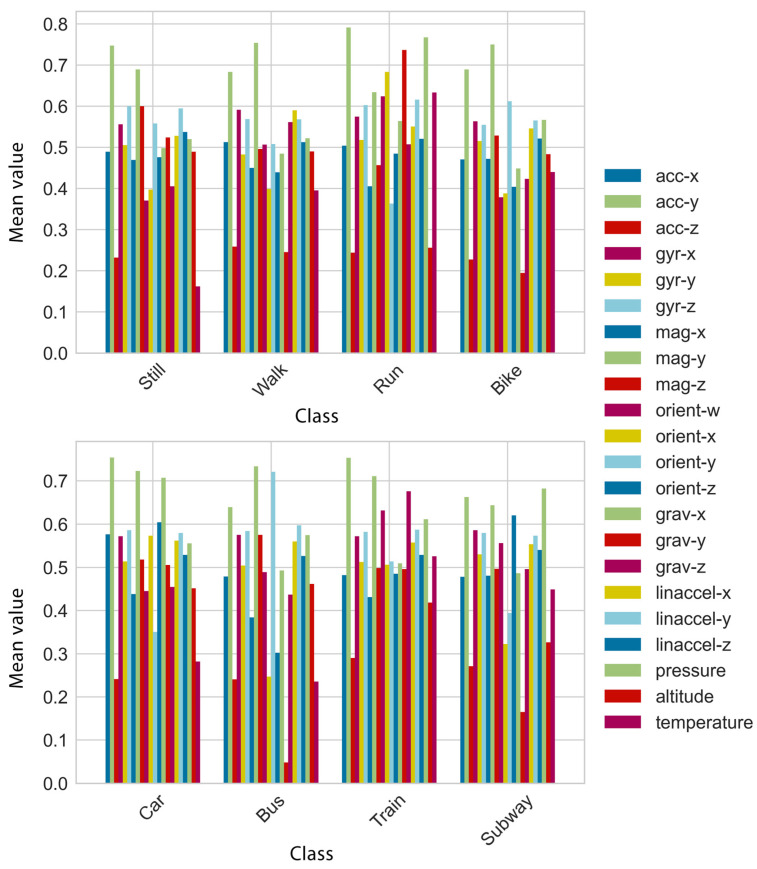
The mean values of features per class.

The dataset was already a supervised learning problem with input and output variables. The first column represents the timestamp of the sample, in milliseconds, while the rest of the columns represent the x, y, and z measurements of the accelerometer (m/s^2^), gyroscope (rad/s), magnetometer (μΤ), gravity (m/s^2^), and linear acceleration (m/s^2^), as well as the w, x, y, and z measurements of orientation, and the ambient pressure (hPa), altitude, and temperature. The mean values of features per class are shown in Figure 1.

**Figure 2:**
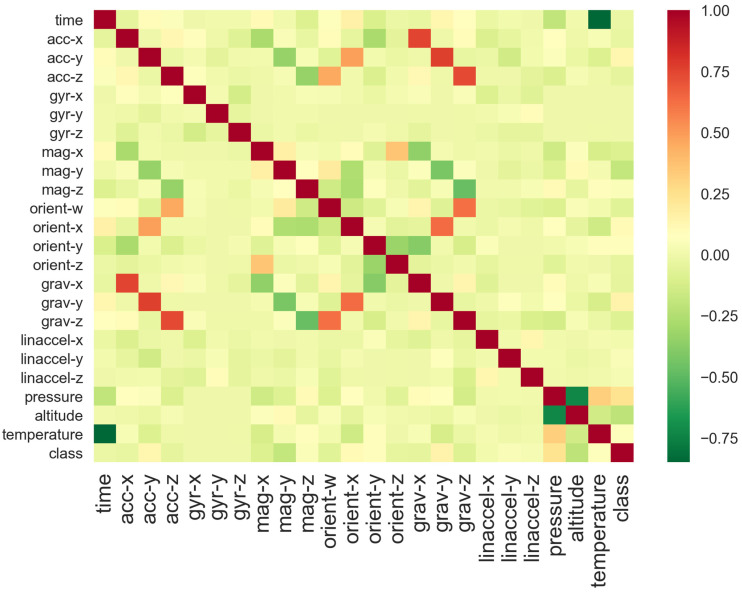
A heat map that visualizes the correlation matrix and shows the correlations between and among the continuous variables.

The correlation between and among the continuous variables, so as to better understand the underlying relationships in the data, is shown in a heat map that visualizes the correlation matrix, in Figure 2. The annotation inside each cell indicates the correlation coefficient of the relationship.

The highest positively correlated features are:

- Acceleration with gravity, which seems reasonable, because the accelerometer outputs the acceleration of the device in three axes by measuring consequent forces applied to the device, and the force of gravity influences this measurement;
- Orientation with gravity, because both are typically derived from the accelerometer.

The highest negatively correlated features are:

- Altitude with pressure;
- Gravity with magnitude.

To get insight—not only on the distribution of a single machine learning variable, but also on the interrelationship between sensor parameters and their impact on each class—the dataset was represented with pair plots using the Seaborn visualization library.

**Figure 3:**
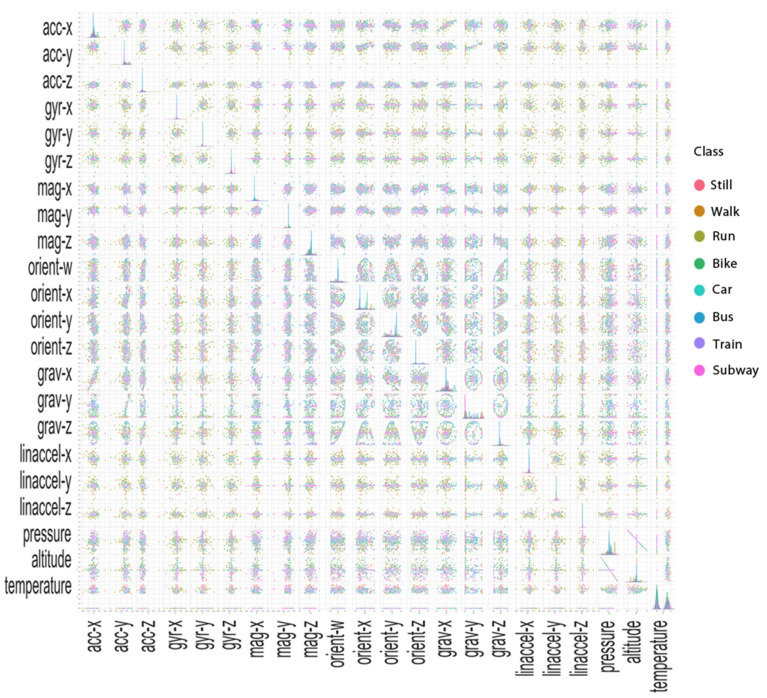
The pair plots show the relationships between all pairs of features per class and in case of a linear relationship, denote the strength of this relationship.

The kde diagrams on the diagonal allow us to see the distribution of a single variable, while the scatterplots on the upper and lower triangles show the relationship (or lack thereof) between two variables. As we can see in Figure 3, the pair plots show the relationships between all pairs of features per class and, in case of a linear relationship, denote the strength of this relationship. The more the dots scatter from the trend line, the weaker the relationship. Strong linear correlations exist between gravity and acceleration (A) and between altitude and pressure (B). Gravity-y and orientation-x (C), as well as gravity-z and orientation-w (C), have medium linear relationships. Gravity and magnitude (D) have a weak linear relationship. The large amount of overlap between classes in the univariate kernel density plots shows that neither feature of the pair alone is able to classify transportation modes very well. The classes are not well separated into clusters based on the sensor measurements. Thus, it becomes very difficult to distinguish one class from another.

### 4.3. Data Preparation

**Figure 4:**
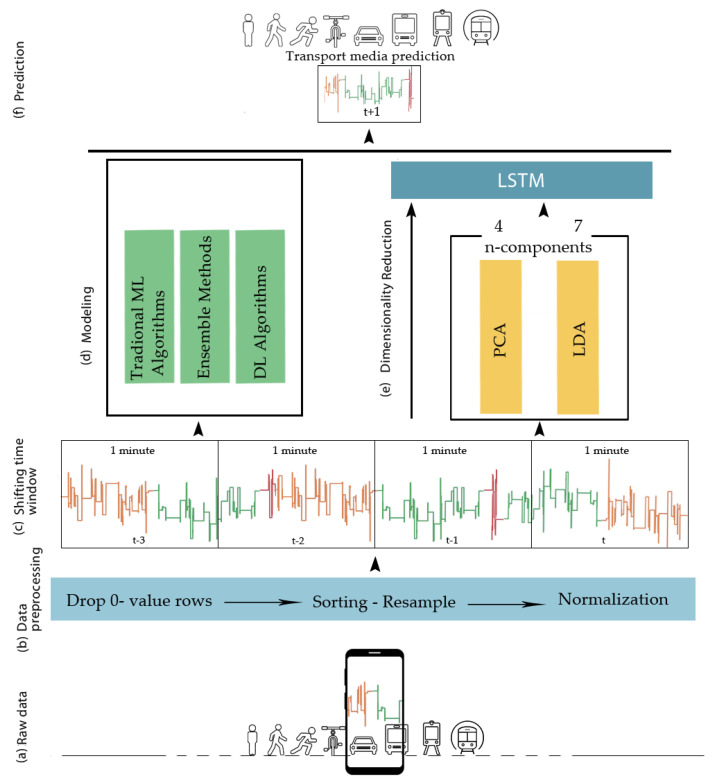
The model structure.

The dataset was already a supervised learning problem with input and output variables. In Figure 4a–c, the data preparation process is depicted. 

For a better performance, two data preprocessing techniques were employed in this work. The first step was to drop rows with class 0 (null class) and sort the column of time values so that the data were sorted by date. The original dataset described the daily sensor measurements for 7 months at various times per day. The dataset was resampled so as to be available at the same frequency that we wanted to make predictions. Downsampling decreased the frequency of the samples from milliseconds to minutes. This makes sense, as switching between transportation modes can be quite frequent. 

The goal was to establish a multivariate LSTM prediction model. The problem was framed so that multiple, recent timesteps could be used to make the prediction for the next timestep. For example, given the current time (t), we wanted to predict the value at the next time in the sequence (t + 1), so we used the current time (t), as well as the one or more prior times (t − 1, t − 2, …, t − n), as input variables. The input features were normalized, as there were differing scales in input values. 

### 4.4. Proposed LSTM Model

First, the dataset was split into training and test sets (80% for training, 20% for testing), and then the training and test sets were split into input and output variables. As depicted in Figure 4d, two approaches were made for constructing the final prediction model. Firstly, the data were fed to LSTM as a whole set of the original 22 features, and then a dimensionality reduction algorithm was used before applying LSTM to the data so as to reduce the dimensions of the training data and positively affect the performance of the model. The inputs were reshaped into the 3D format expected by LSTM. The LSTM model selected through the Bayesian optimization process was defined with 64 neurons in the first hidden layer, and an output layer with a softmax activation function and 9 output values. Only one layer of LSTM was used, but different numbers of LSTM cells—such as 128, 256, 512, and 1024, which are the most used numbers in the literature—were tested. Categorical cross-entropy was used as a method for error calculation and, in order to update the weights of our neural network, the Adam optimizer was used, with a learning rate of 0.001. Additionally, dropout was implemented to randomly drop 20% of units from the network. To reduce underfitting and improve model performance, the network was fitted for a different number of epochs and various batch sizes, while a 10-fold cross-validation was used to overcome the limitation of dataset size and prevent overfitting.

Each experimental scenario was run 10 times with the same parameters so as to obtain an average outcome. Various tests were conducted concerning resampling, LSTM model (number of cells, dropout), epochs, and batch size variances.

The model learned better after each epoch. However, it started to memorize with the increasing number of epochs. As the batch size value decreased, the complication of the model increased and gave better results, but after a certain time there was no significant improvement.

### 4.5. Experimental Results

In this subsection, we evaluate the performance of the proposed optimized LSTM model. Several traditional machine learning algorithms, ensemble methods, and deep learning models were used to determine transportation modes as a benchmark to the LSTM model, as shown in Figure 4e:

- Traditional machine learning algorithms: logistic regression (LR), linear discriminant analysis (LDA), k-nearest neighbor (kNN), classification and regression tree (CART), naive Bayes (NB), multilayer perceptrons (MLPs);
- Ensemble algorithms: random forest (RF, bagging algorithm), AdaBoost, XGBoost, (boosting algorithms), bagging (bootstrap aggregating), extra trees (extremely randomized trees), voting (hard or soft voting);
- Deep learning algorithms: convolutional neural network (CNN), bidirectional long short-term memory networks (Bi-LSTM).

The hyperparameters of all models were optimized, and are summarized in Table 2, Table 3 and Table 4. 

In order to evaluate the performance of different classification models, among several metrics used in this study—i.e., accuracy, precision, recall, F1-score, and confusion matrix—weighted F1-score was selected to be the most representative, as it computes the F1-score for each label, and returns the average, considering the proportion of each label in the dataset. 

For forecasting, three different cases were used, i.e., 1-before, 2-before, and 3-before, meaning that 1, 2, and 3 min before were taken into consideration, respectively, so as to predict the transportation media used in the next minute. 

As presented in Table 5, all traditional ML algorithms had similar F1-scores in all three cases, apart from ANN, which performed better when prediction took 3 previous minutes into consideration. k-NN performed best, achieving 98% in all three cases.

**Figure 5:**
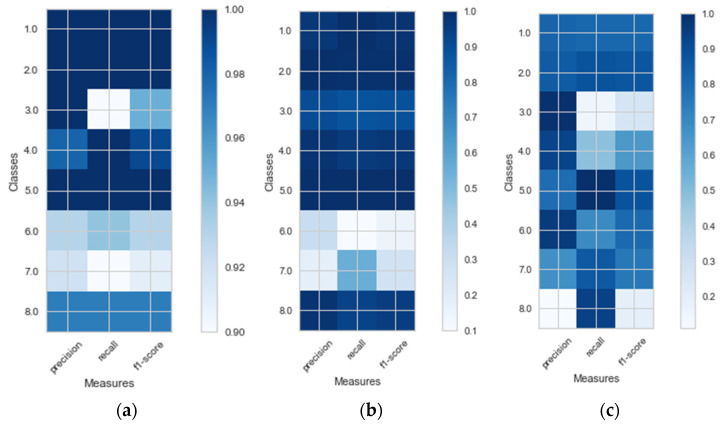
Classification reports for (**a**) k-NN in the 1-before case, (**b**) ANN in the 3-before case, and (**c**) ANN in the 1-before case.

As Figure 5 shows, k-NN classified all classes the best, whereas ANN showed a better prediction in the case of 3-before for all classes, except for run and subway.

The ensemble methods performed better than the traditional ML algorithms, as presented in Table 6. AdaBoost in the case of 3-before, and hard voting in the 1-before and 2-before cases, reached 98% and 99% F1-scores, respectively. 

**Figure 6:**
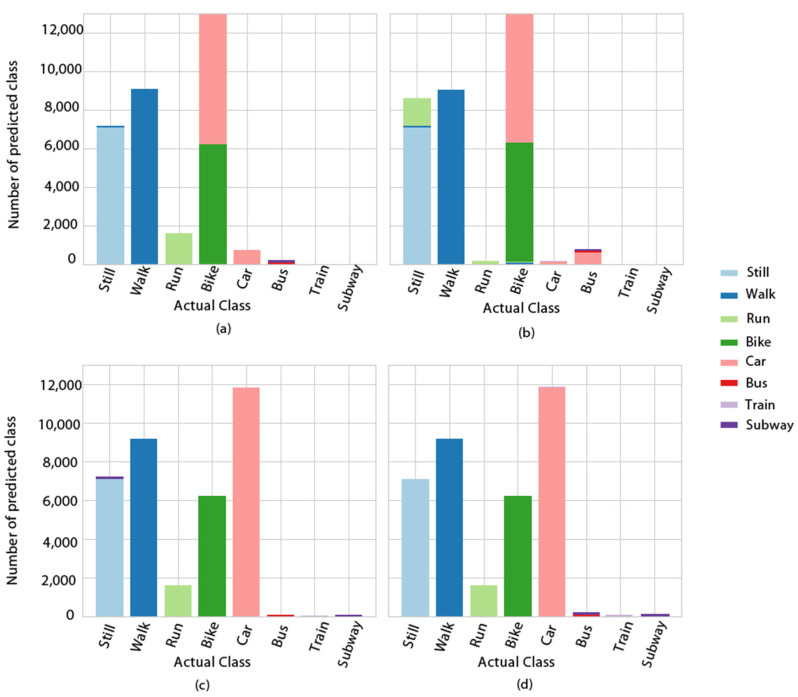
Class prediction error for (**a**) AdaBoost (random forest) in the 1-before case; (**b**) extra trees in the 1-before case; (**c**) hard voting in the 2-before case; and (**d**) AdaBoost (random forest) in the 3-before case.

**Figure 7:**
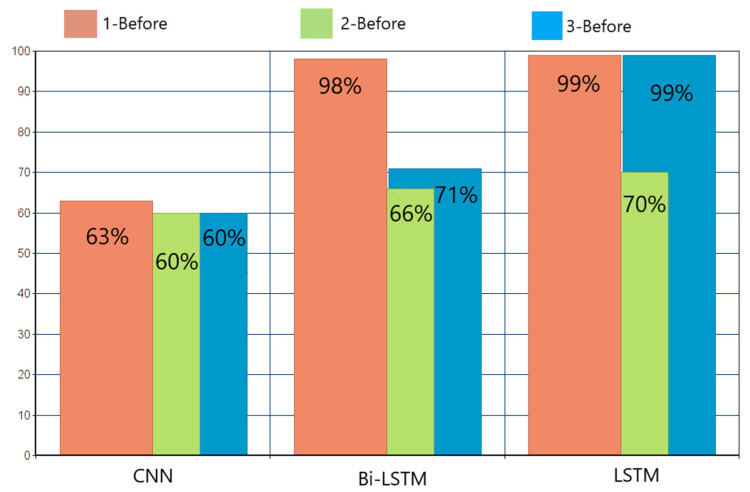
Deep learning algorithms’ F1-scores in the 1-, 2-, and 3-before cases.

**Figure 8:**
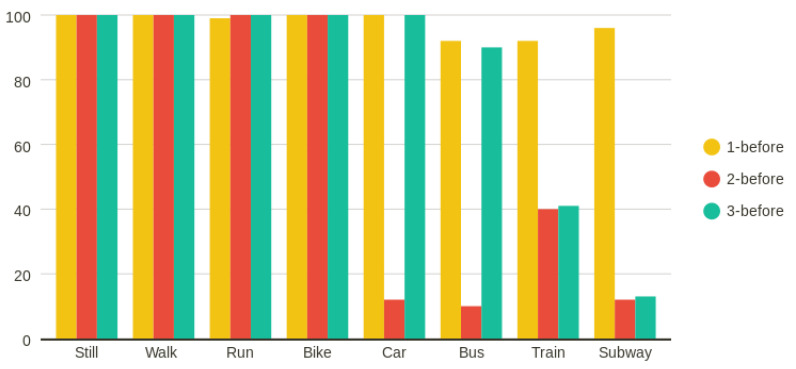
LSTM’s F1-score for each class in the 1-, 2-, and 3-before cases.

The ensemble methods that achieved less than 70% F1-scores had difficulty in accurately predicting the car and bike classes, as presented for example in Figure 6a,b, where AdaBoost and extra trees in the 1-before case prediction per class are presented. In Figure 6c,d, the same results are shown for the two cases that achieved the best results—hard voting in the 2-before case, and AdaBoost in the 3-before case—where the prediction was almost perfect. DL methods performed as shown in Figure 7. Bi-LSTM performed well, achieving a 98% F1-score only in the 1-before case, whereas LSTM in both the 1-before and 3-before cases reached 99%. In Figure 8, the LSTM F1-scores for each class are presented. All classes were predicted better in the 1-before case and, in general, among all classes, the motorized media were more difficult to predict accurately—especially in the 2-before case. 

The training times for each algorithm achieving an F1-score of more than 95% are presented in Table 7. XGBoost was the fastest, followed by the deep learning algorithms—namely, Bi-LSTM and LSTM—which, despite their complex structures, performed faster than the others in all three cases.

**Figure 9:**
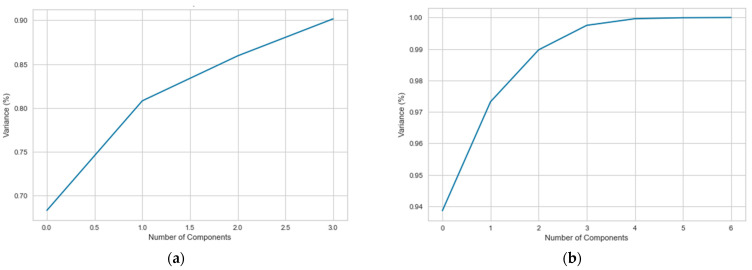
Number of principal components for the (**a**) PCA algorithm and (**b**) LDA algorithm.

To reduce the number of input features, two dimensionality reduction techniques were applied prior to the LSTM model: principal component analysis (PCA), and linear discriminant analysis (LDA). Several n-components’ values were tested to accomplish the best performance of the model. According to Figure 9, for the implementation of PCA, four principal components were selected so as to preserve over 90% of the total variance of the data, and seven principal components were finally selected for LDA.

**Figure 10:**
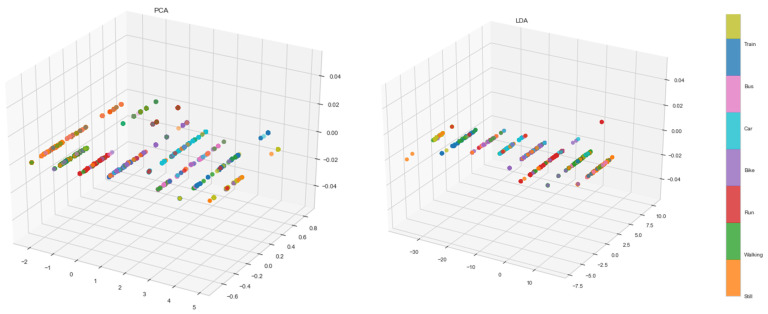
Visualization of the first three principal components of PCA in the 1-before case.

In Table 8, the results of these two techniques are presented concerning F1-scores and accuracy metrics. It is clear that PCA had better results than LDA, and made LSTM perform better in all three cases. PCA worked best in that direction, achieving an increase in F1-score from 99.5 to 99.8%. In the 3D scatterplot in Figure 10, we see that PCA’s three components hold more information than LDA’s, especially for specific classes—i.e., still, walk, run, car—but clearly not enough to set all of them apart. 

**Figure 11:**
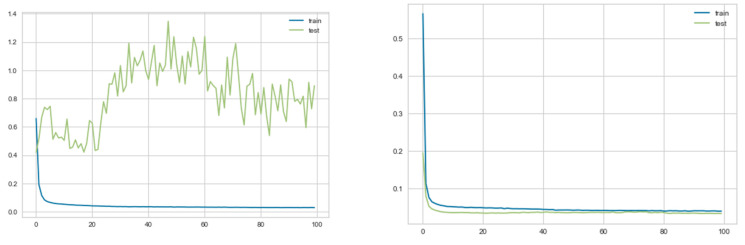
Learning curves indicating loss for the 2-before case, before and after applying PCA.

A plot of learning curves is shown in Figure 11, indicating loss for the 2-before case, both before and after applying PCA. With PCA, the training and validation loss decreased to a point of stability, with a minimal generalization gap between the two final loss values, showing a well-fitted model.

**Figure 12:**
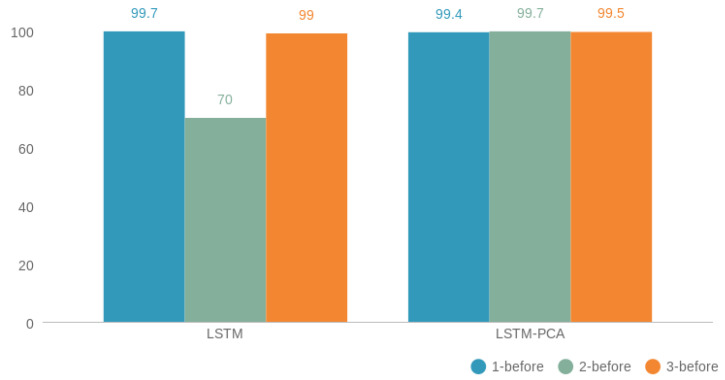
LSTM’s performance before and after applying PCA for the 1-, 2-, and 3-before cases.

Figure 12 depicts LSTM’s performance before and after reducing the dimensionality of the data. With PCA, the performance was improved in the 2-before and 3-before cases, but in 1-before it decreased by ~0.3%. 

**Figure 13:**
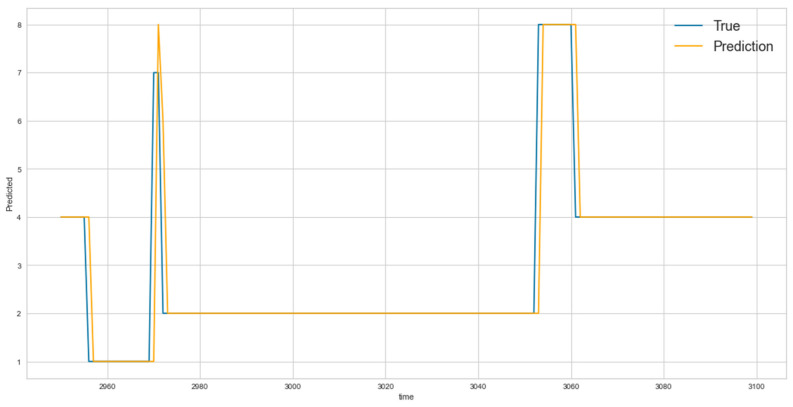
Predicted and actual average values of all 1-, 2-, and 3-before cases for LSTM after applying the PCA algorithm.

In Figure 13, the blue line represents the average values of the actual transportation media used for all 1-, 2-, and 3-before cases, while the yellow line represents the predicted values. It is clear that LSTM was able to capture the overall trend.

**Figure 14:**
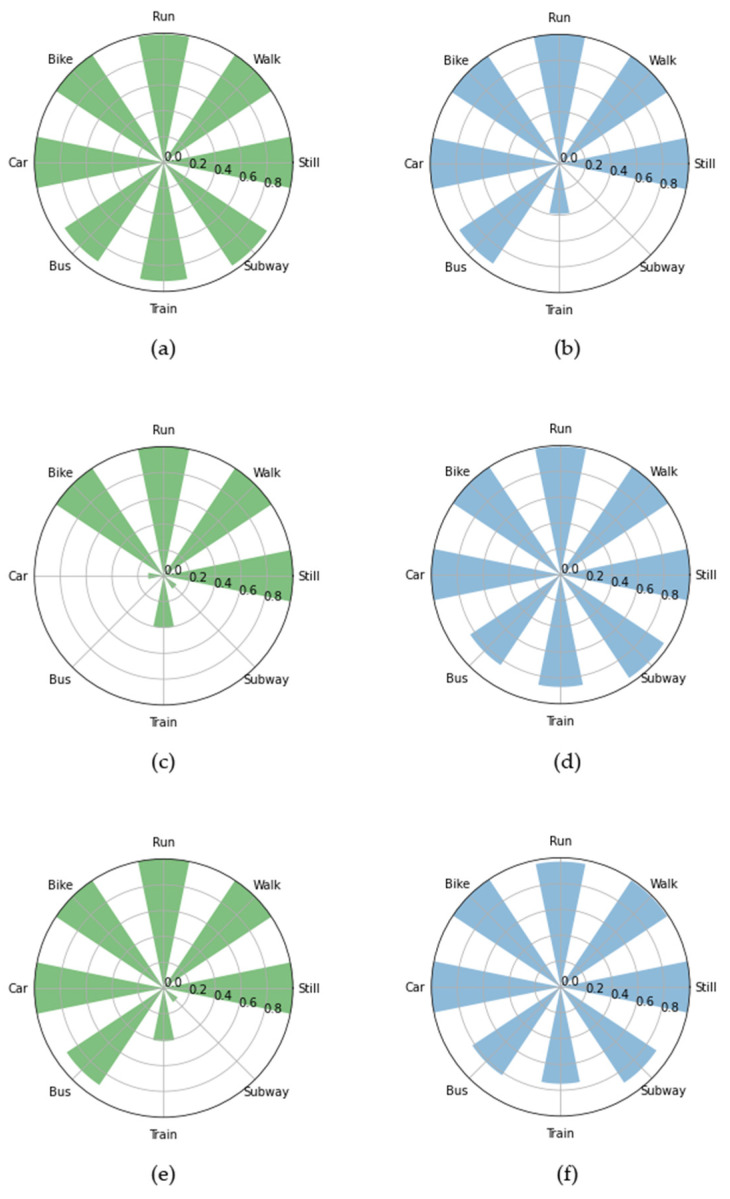
The classes predicted before (**a**,**c**,**e**) and after (**b**,**d**,**f**) applying PCA for the 1-, 2-, and 3-before cases.

The classes predicted before and after applying PCA are presented in Figure 14. In the 1-before case, the train and subway classes were predicted worse after dimensionality reduction, whereas with PCA all classes were predicted better in the other two cases.

**Figure 15:**
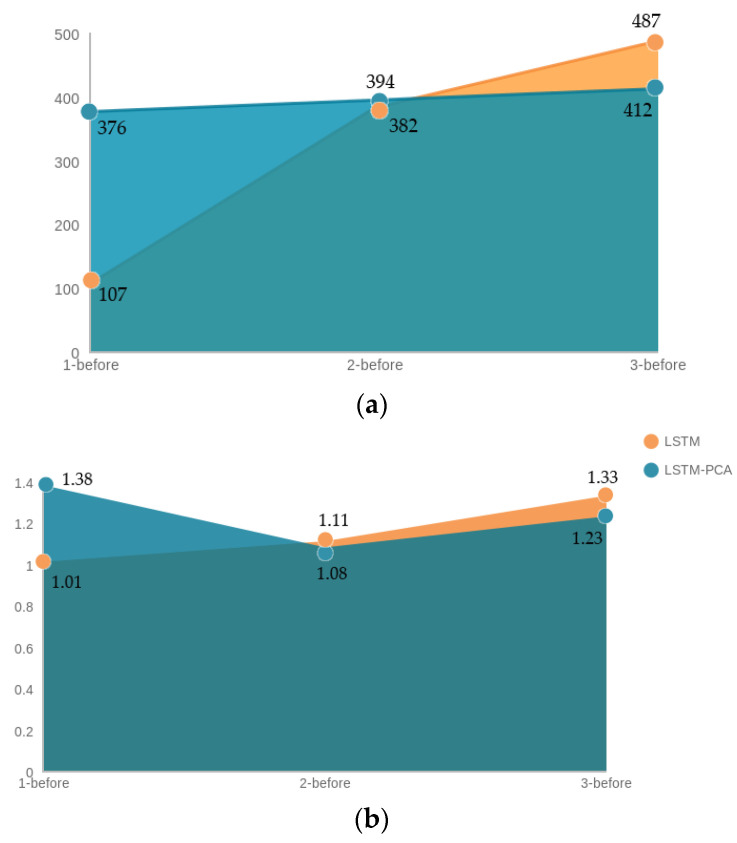
(**a**) Training time and (**b**) prediction time before and after applying PCA to LSTM.

In terms of time, according to Figure 15, LSTM–PCA had an average training time of 394 s in all three cases, while LSTM without dimensionality reduction had an average training time of 325 s. Both models’ testing times had similar average values, at 1.23 and 1.15 s, respectively.

## 5. Conclusions

In this work, we scrutinized the effectiveness of several machine learning methods used for transportation mode detection based on multimodal smartphone sensor data. Our proposed Bayesian-optimized LSTM model can recognize eight transportation modes with 99.7% accuracy, and significantly outperforms other state-of-the-art methods. Apart from the superior recognition rates attained by our proposed model, a few additional interesting conclusions can be drawn from our evaluation. 

Among traditional ML algorithms, k-NN and ANN (MLP) performed better in all three cases and in the 3-before case, respectively. The transport media that were predicted more accurately were the non-motorized rather than the motorized ones. The experiments also showed that ensemble methods performed better than the traditional ML algorithms—especially AdaBoost (random forest) in the 3-before case and hard voting (random forest, SVC, decision trees) in the 1- and 2-before cases. Among these methods, the ones that performed worse had difficulty in distinguishing cars from bikes, cars from buses, buses from subway, and still from walking. 

Among deep learning models, the best results were obtained by the proposed optimized LSTM only in the 1-before and 3-before cases. LSTM managed to accurately predict all transport media in the 1-before case, whereas in the 3-before case it did not perform well for train or subway. 

After dimensionality reduction with PCA applied to the dataset, LSTM achieved a high F1-scores for all three cases, with the highest F1-score of 99.7%. The classes were predicted with almost 100% accuracy in the 2-before and 3-before cases. On the other hand, in the 1-before case, there was a better prediction for all classes without applying PCA. Both LSTM and PCA–LSTM showed an increase in all three cases, but applying PCA before LSTM caused a smoother change in time values. In general, the training time was not long, but it is clear from Figure 15 that PCA–LSTM took less time in the 1-before case and more in the 3-before case compared to LSTM. Similarly, in terms of prediction time, it took longer for LSTM to predict the transport media from 3 min before, and less time from 1 min before. In the 2-before case, both training and prediction times had similar values.

Despite the good results achieved in this study, there were also some limitations. Firstly, the limited number of annotated data—especially for specific classes—along with the great computational complexity due to the large number of model parameters, resulted in the model finding it difficult to distinguish certain classes, such as car and bike. Secondly, the fact that not all people own a smartphone (e.g., residents of developing countries, elderly people) would make it impossible to detect the transportation mode they use in this way; thus, alternative methods should be explored.

The automatic recognition of transportation modes, which was described in this work using data from the sensors of a modern mobile device, can be further used for valuable support in various intelligent transportation system (ITS) applications, such as driving behavior monitoring, human activity monitoring, urban transportation planning, road environment and traffic prediction, health monitoring, etc. Such an application, for example, could be particularly useful to internet service providers so as to allocate their resources effectively and provide a more personalized and enhanced experience to the citizens. 

Future directions of our work include the following: using a larger and more balanced dataset to improve the model and attain a better and more accurate distinction between all classes; investigating alternative ways to detect transportation mode without smartphones; investigating approaches based on transformer neural networks, so as to be immediately able to process larger datasets without the restriction of processing sequential data and, secondly, to reduce training time so as to result in a more accurate and efficient model; and investigating methods for increased explainability of the system outcome (explainable artificial intelligence—XAI), while maintaining a high level of learning performance/prediction accuracy, so as to develop models that are both robust in terms of accuracy and notably interpretable during all of the stages of development. 

## Figures and Tables

**Table 1:** Samples per class.

Class	Samples
Still	19,085
Walk	46,987
Run	39,814
Bike	43,988
Car	26,268
Bus	3861
Train	623
Subway	693

**Table 2:** The parameters of traditional ML algorithms.

	Ada-Boost	Bagging	Extra Trees	XG Boosting	Voting
n_estimators	1000	150	150		-
Base estimators	Random forest classifier	Decision tree classifier	-		RF, SVC, DT
Number of trees	-	500	-		-
Max_features	-	-	5		-
RF n-estimators	-	-	-		50
SVC parameters	-	-	-		default

**Table 3:** The parameters of ensemble methods.

	LR	LDA	k-NN	NN	RF	CART
Multiclass	One-vs-rest	-	-	-	-	-
K value			5	-	-	-
Classifier	-	-	-	MLP	-	-
Layers				100 hidden		
n_estimators	-	-	-	-	20	-
Max depth	-	-	-	-	5	-
Max tree depth	-	-	-	-	-	None
Solver	-	Singular value decomposition (svd)	-	-	-	-

**Table 4:** The parameters of DL methods.

	CNN	Bi-LSTM	LSTM
Conv1D layer	2	-	-
Activation	Relu	-	-
Layers	MaxPooling Flatten	-	-
Number of neurons in the first layer	8	8	64
Output values	9	9	9
Optimizer	Adam	Adam	Adam
Dropout	0.1	0.1	0.2
Learning rate	0.001	0.001	0.001
Error calculation	Categorical cross-entropy	Categorical cross-entropy	Categorical cross-entropy
Dense layer	2 (50,9)	1(9)	1 (9)
Activation	ReLU, softmax	Softmax	Softmax

**Table 5:** F1-scores for cases 1-, 2-, and 3-before of applying traditional ML algorithms.

	LR	LDA	k-NN	CART	NB	ANN	RF
1-before	70	87	98	92	92	79	87
2-before	68	87	98	91	92	86	88
3-before	68	87	98	92	89	98	87

**Table 6:** F1-scores for cases 1-, 2-, and 3-before of applying traditional ensemble methods.

	AdaBoost (RF)	Bagging	Extra Trees	XG Boosting	Hard Voting
1-before	62	92	53	98	98
2-before	69	92	50	98	99
3-before	99	92	82	98	95

**Table 7:** Training time (in seconds) for the algorithms achieving F1-scores of more than 95% in the 1-, 2-, and 3-before cases.

	1-Before	2-Before	3-Before
kNN	128	323	346
ANN			2264
AdaBoost (RF)			172
Bagging	230	453	704
XGBoost	33	53	78
Hard voting	433	761	
Bi-LSTM	108		
LSTM	107		500

**Table 8:** F1-score/accuracy metrics and number of n-components in the 1-, 2-, and 3-before cases for LSTM after applying PCA and LDA algorithms.

	PCA-LSTM	LDA-LSTM
1-before	99.8/99.5 (4 n-components)	70/68 (7 n-components)
2-before	99.7/99.7 (5 n-components)	97/99 (7 n-components)
3-before	99.5/99.5 (15 n-components)	97/99.7 (7 n-components)

## Data Availability

Publicly available datasets were analyzed in this study. This data can be found here: http://www.shl-dataset.org.
